# Gallbladder Volvulus: A Review

**DOI:** 10.7759/cureus.23362

**Published:** 2022-03-21

**Authors:** Nattawut Keeratibharat, Jirapa Chansangrat

**Affiliations:** 1 School of Surgery, Institute of Medicine, Suranaree University of Technology, Nakhon Ratchasima, THA; 2 School of Radiology, Institute of Medicine, Suranaree University of Technology, Nakhon Ratchasima, THA

**Keywords:** acute abdomen, acute cholecystitis, gallbladder torsion, gallbladder volvulus, gallbladder

## Abstract

Gallbladder volvulus is an uncommon condition that mostly affects older women. The cause of gallbladder volvulus is unknown, although intraoperative evidence of a floating gallbladder with a twisting of its pedicle, resulting in gallbladder ischemia, may lead to subsequent complications. Gallbladder volvulus symptoms are similar to acute cholecystitis, leading to delayed diagnosis and treatment. Early detection and prompt surgical intervention are critical for reducing morbidity and mortality. Even though numerous case reports have been published since 1898, gallbladder volvulus remains challenging to diagnose preoperatively. As a result, a high level of suspicion is required to prompt cholecystectomy and avoid further complications. We review the etiology, pathophysiology, clinical manifestation, diagnostic strategies, and treatment of this disease.

## Introduction and background

**Figure 1:**
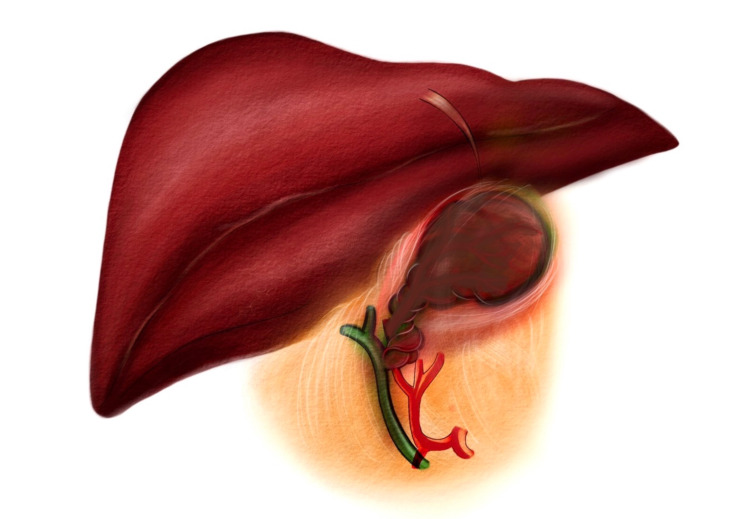
Twisted gallbladder along its axis, resulting in gallbladder volvulus and ischemia Image credit: Dr. Jirapa Chansangrat

Gallbladder volvulus is an uncommon disorder occurring when the gallbladder twists along its axis (Figure 1), with only approximately 500 occurrences documented in the English literature [1-2]. Preoperative diagnosis is difficult and possible in less than 10% of patients [3]. Gallbladder volvulus has clinical and physical manifestations similar to acute cholecystitis, making it challenging to distinguish [4]. Because acute cholecystitis typically does not necessitate prompt surgical intervention, emergent cholecystectomy is the preferred treatment in this case. Delays in treatment may result in increased septicemia due to gallbladder ischemia, necrosis, and perforation, which can be fatal [5]. Pathophysiology, risk factors, and clinical information, including recent preoperative investigation and treatment, are summarized in this review.

## Review

Embryology of the liver and biliary tract

The liver comprises two primordia: the liver diverticulum and the septum transversum. The liver diverticulum develops from endodermal cells in the foregut and extends cranioventrally into the septum transversum. The process of generating an organ bud happens between 26 and 32 days of gestation. At the 5-mm stage, the diverticulum has a solid cranial hepatic component and a hollow caudal portion. The intrahepatic bile ducts form from primitive hepatocytes that form around portal vein branches. The extrahepatic bile ducts and gallbladder emerge from the ventral foregut near the liver and pancreas. Abnormalities in the process of embryonic bud migration result in a variety of gallbladder malposition. The cause of the left-sided gallbladder is most likely the migration of embryonic bud from hepatic diverticula to the left rather than the right [6]. A caudal bud that extends beyond the cranial bud may result in an intrahepatic gallbladder. If the caudal bud moves slower than the cranial bud, the gallbladder will be covered by the peritoneum and suspended from the liver by mesentery, resulting in a floating gallbladder [7].

Pathophysiology and risk factors

The exact causes of gallbladder volvulus are unknown. Several hypotheses have been proposed. One of the most significant risk factors is gallbladder anatomical variation. The gallbladder usually attaches to the liver through a gallbladder fossa on the right lobe. The visceral peritoneum covers it anteriorly and blends it with the liver capsule. The visceral peritoneum covers the posterior part of the abdomen to the tip. These structures support the gallbladder and keep it from wandering. The Gross classification distinguished two anatomical variants of the mesentery resulting from embryological anomalies of gallbladder migration, resulting in a congenitally floating gallbladder. The mesentery suspends the floating gallbladder from the liver surface and is only covered by the visceral peritoneum; type A corresponds to a long and wide mesentery that supports the gallbladder and cystic duct; type B includes an incomplete or absent mesentery that only connects the cystic duct to the liver [8]. The changed anatomy affected gallbladder movement, which increased the likelihood of volvulus. This anatomical variance is estimated to be around 1.3% [9].

Another crucial reason is the absence of supportive tissue around the gallbladder fossa, such as fat, connective tissue, and neighboring liver parenchymal volume, which results in a free-floating gallbladder [10-11]. These disorders are risk factors for aging, excessive weight loss, and liver shrinkage [12-13]. Because it permits the gallbladder to position vertically, kyphoscoliosis increases the risk of gallbladder volvulus [14]. The cystic artery and tortuous cystic duct have also been proposed as cofactors, possibly acting as fulcrums for the gallbladder to twist [15-16]. Variation and altered anatomical structures and a dynamic process are proposed as provoke factors for the occurrence of gallbladder volvulus. A sudden shift in body position, a blunt abdominal injury, and excessive stomach or bowel movement near the gallbladder are examples of these processes.

Increased cholecystokinin is thought to be a hormonal provocation factor that causes gallbladder contraction and, as a result, gallbladder volvulus [17]. Gallstones were thought to increase the risk of gallbladder volvulus by increasing the weight of the gallbladder [14,18]. However, according to a large series investigation, At the time of diagnosis, 50-80% of people with gallbladder volvulus do not have a gallstone [3,19]. There is currently no evidence of a correlation between gallstones and gallbladder volvulus. There was no significant correlation between torsion direction (clockwise or counterclockwise) and torsion degree (180°-incomplete torsion, >180°-complete torsion) [5].

To summarize, the risk of gallbladder volvulus due to hypermobile "floating" gallbladder could be caused by congenital variation, loss of supporting structures, or anatomical triggering in twisting motion [20].

Epidemiology

Patients' ages vary from three to 96 years old, according to published research, with two peaks: an aging patient group (84%) aged more than 70 years and a pediatric patient group (16 %) aged less than 18 years. With a 5:1 ratio, females outnumber males in the geriatric population while males outnumber females in the pediatric population [3,5,21]. Because of the predominance of the elderly age group, 76.2% of female patients are diagnosed while 23.8% of male patients are diagnosed. 

The congenital anatomical variant causes gallbladder volvulus in pediatric cases, consistent with the anatomical variation's rare incidence. On the other hand, adult gallbladder volvulus seems to be caused by physiological changes associated with aging.

Clinical presentation

Patients with gallbladder volvulus often report nonspecific symptoms, such as abdominal ache (100%), nausea and vomiting (52.7%), palpable lump (32.6%), fever (31.8%), and jaundice (31.8%), according to an analysis of 245 cases (0.8% ) [3]. The minority of patients who present with jaundice differ from most patients diagnosed with typical acute cholecystitis. 

Lau et al. first described the triad of triads used to identify potential gallbladder volvulus, as shown in Table 1 [20].

**Table 1:** The triad of triads used to identify potential gallbladder volvulus

Appearance	Symptom	Physical examination
Elderly (usually female)	Sudden onset	Nontoxic presentation
Thin habitus	Right upper quadrant pain	Palpable right upper quadrant mass
Spinal deformity	Early emesis	Pulse-temperature discrepancy

Biliary colic may be misdiagnosed as incomplete gallbladder torsion. Torsion of the gallbladder ultimately impairs artery flow, leading to gallbladder ischemia, necrosis, gangrene, and perforation [10,15,22].

Diagnostic strategies

Serologic tests might be imprecise and typically mimic an acute inflammatory illness [23]. In 55% of patients, inflammatory indicators, such as C-reactive protein (CRP) and white blood cell count, are elevated. In about 85% of patients, liver function tests are within normal limits [21]. Imaging has shown to be essential in identifying gallbladder volvulus prior to surgery.

Ultrasound

Ultrasound can be used to visualize gallbladder morphology and surrounding structures [15,22]. The gallbladder wall should be markedly thickened in gallbladder volvulus, which cannot be distinguished from acute cholecystitis. One study described a continuous hypoechoic zone that exists between the two echogenic layers of the wall. This observation implies edematous wall changes caused by a mix of venous and lymphatic stasis [24].

Specific ultrasound findings in several publications indicate that the gallbladder is positioned outside the fossa and inferior to the liver and that it is attached to the liver via a conical structure corresponding to the twisted pedicle, but these findings are unusual [25-28]. Doppler ultrasound can help diagnose by demonstrating interrupted blood flow in the cystic pedicle [14]. According to one study, the torsive gallbladder had no blood flow, but the cystic artery along the wall could be seen in acute cholecystitis [29].

Computed Tomography

**Figure 2:**
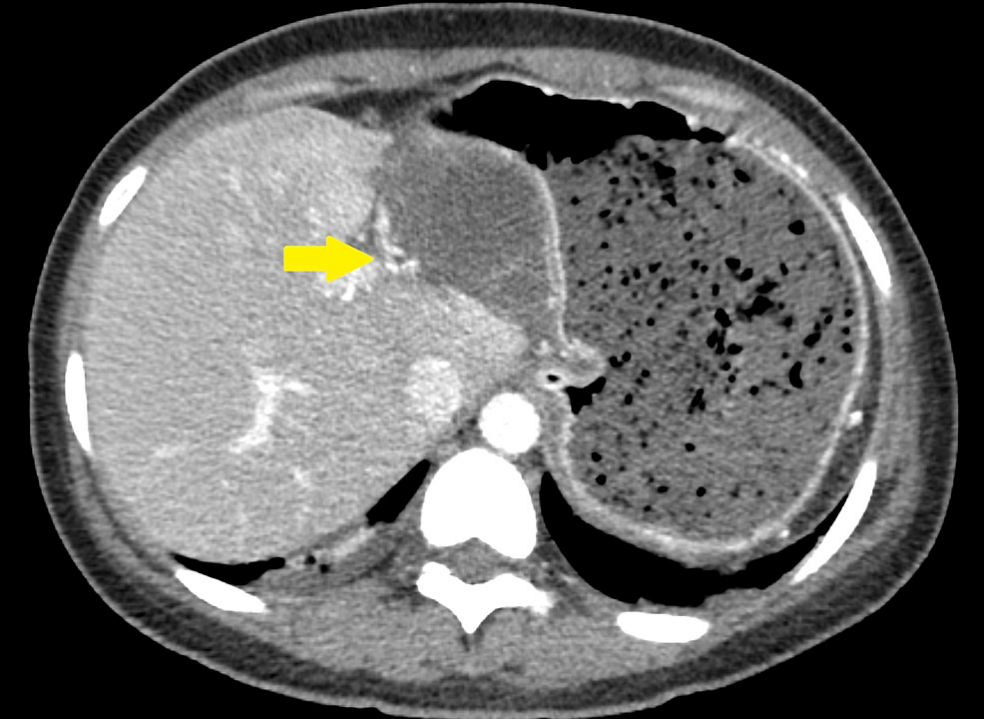
Axial CT scan in arterial phase shows twisted gallbladder vascular pedicle (arrow)

**Figure 3:**
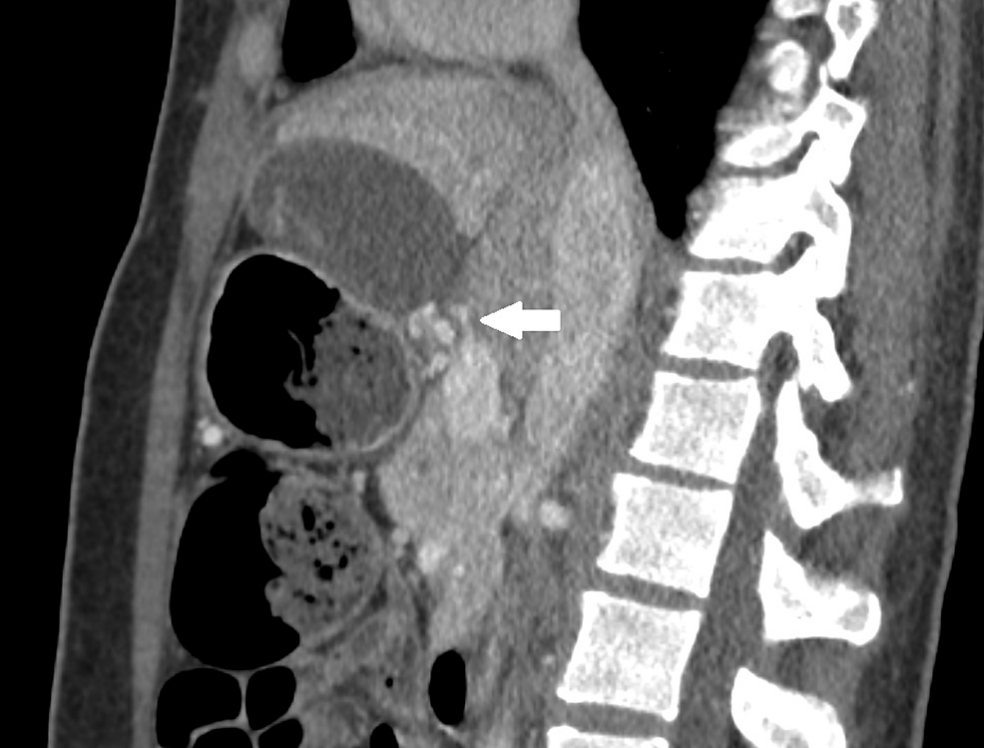
Sagittal CT scan in arterial phase shows twisted gallbladder vascular pedicle with swirl appearance (arrow)

**Figure 4:**
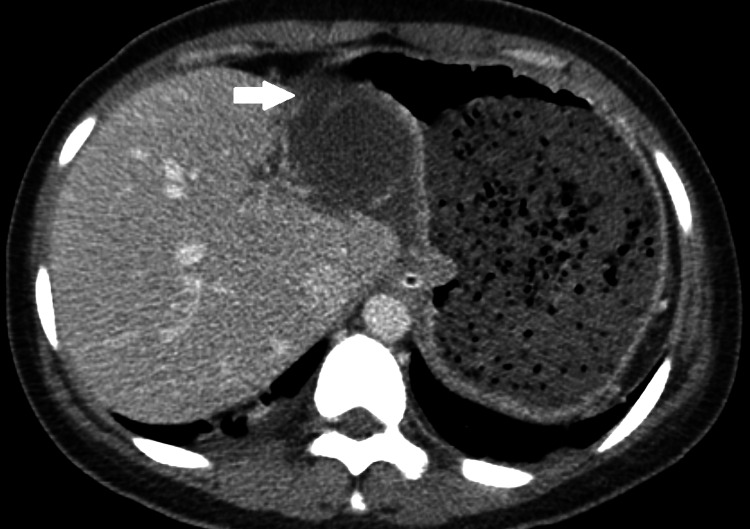
Axial CT scan in venous phase shows diminished enhancement of the gallbladder wall (arrow)

On computed tomography, the imaging characteristics of gallbladder volvulus are not unique [30]. As diagnostic criteria, collection in the gallbladder fossa, gallbladder outside its anatomical fossa, vertical to horizontal rotation of the gallbladder's axis, twist along the gallbladder's vascular pedicle with swirl appearance (Figure 2 and Figure 3), inflammatory characteristics of the gallbladder wall, or abrupt tapering of the cystic duct have all been proposed [11,31]. Reduced enhancement of the gallbladder wall frequently indicates parietal ischemia (Figure 4) [32]. According to some reports, gallbladder volvulus causes more gallbladder distention than acute cholecystitis. Due to wall scarring from prior episodes of inflammation, gallbladder distension in acute cholecystitis is unlikely to be severe [30].

Nuclear Medicine Imaging

The hepatobiliary iminodiacetic acid (HIDA) scan may reveal a "Bull's eye" look, indicating radioactive tracer deposition within the gallbladder [19,21].

Magnetic Resonance Imaging

On T1-weighted images, magnetic resonance imaging may show a high signal intensity within the gallbladder wall, indicating necrosis and hemorrhage [33].

Magnetic Resonance Cholangiopancreatography

Because of its advantage in revealing the cystic duct, magnetic resonance cholangiopancreatography (MRCP) is an excellent tool for detecting gallbladder volvulus [5,21]. There are four imaging characteristics associated with gallbladder volvulus: 1. v-shaped distortion of the extrahepatic bile ducts caused by cystic duct traction; 2. tapering and twisting interruption of the cystic duct; 3. distended, enlarged gallbladder deviated toward the midline; and 4. the gallbladder, extrahepatic bile ducts, and cystic duct may show different intensities [34].

Treatment

Medical Treatment

Gallbladder volvulus can mimic acute cholecystitis and is more common in the elderly. As a result, it is frequently possible to mistreat gallbladder volvulus patients with antibiotics. Conservative and antibiotic treatment may be successful in typical acute cholecystitis. However, this is unlike the ischemic process of gallbladder volvulus, which does not improve with conservative treatment. Furthermore, board-spectrum antibiotic treatment is likely to result in no response to therapy and poor outcomes [3,26,35]. Despite the lack of research on microorganisms and effective antibiotics in gallbladder volvulus, we believe that initial antibiotic therapy with a single broad-spectrum antibiotic is sufficient.

Percutaneous Gallbladder Drainage

Acute calculus or acalculous cholecystitis are often managed non-operatively, with percutaneous gallbladder drainage as the initial treatment and sometimes definitive treatment. In contrast, percutaneous gallbladder drainage is not a recommended option in the treatment of gallbladder volvulus since the disorder is not mainly an infectious process, and the procedure does not treat the underlying pathology [36]. Furthermore, bile leakage and bile peritonitis after transhepatic cholecystostomy would be increased due to the minimal attachment of the gallbladder to the fossa and the necrotic gallbladder wall [3,37]. Nonetheless, no case series or cohort studies on the clinical outcome of percutaneous gallbladder drainage in cases with gallbladder volvulus have been published.

Endoscopic Treatment

There have been few studies on therapeutic endoscopy with endoscopic retrograde cholangiopancreatography (ERCP). There are only two reports of effective endoscopic treatment for gallbladder volvulus in four cases [14,38].

Surgery

In a prior study, 99% of patients who had surgery had gallbladder volvulus treated with cholecystectomy [21]. Cholecystectomy can be performed either laparotomy or laparoscopy. The procedure is feasible, safe, and applicable to any type of gallbladder volvulus. There is no study that compares the outcomes of laparoscopic and laparotomy surgery. Laparoscopy, on the other hand, revealed the advantages of minimally invasive surgery: less pain, a lower rate of postoperative complication, and a shorter duration of stay [39-40]. When possible, we believe that laparoscopy should be the first approach of surgery for gallbladder volvulus (Video 1).

**Video 1:** Laparoscopic cholecystectomy in gallbladder volvulus Video credit: Dr. Nattawut Keeratibharat

Decompression, detorsion, and gallbladder removal are the three essential steps of cholecystectomy in gallbladder volvulus [22]. We fully support this principle because the critical view of safety must be well established to avoid iatrogenic bile duct and vascular injury. Because the twisted gallbladder might tent the CBD, making it prone to damage, meticulous dissection is crucial [41]. However, there was a considerable concern of reperfusion damage from toxin release to the systemic effect following gallbladder detorsion in the case of gallbladder necrosis [42]. Still, no reports about reperfusion injury after gallbladder detorsion in the necrotic condition have been published.

Outcomes

Misdiagnosed gallbladder volvulus progresses to ischemia, necrosis, gangrene, perforation, peritonitis, multiorgan failure, and death [32]. Morbidity and mortality were observed in 16% and 6% of the cases, respectively [21]. In the last three decades, all of the deaths have occurred in elderly patients, and none of them were diagnosed with gallbladder volvulus prior to surgery. Furthermore, a delay of more than two days was associated with increases in mortality [43]. Although their data is not recent, the association between delayed surgery and poorer results is plausible.

Discussion

Volvulus of the gallbladder is a rare occurrence. Preoperative diagnosis using clinical data and imaging is frequently confused with acute cholecystitis. Knowing the predisposing factors and characteristics of the patient at risk would allow the condition to be included in the differential diagnosis. In the elderly, with the major peak incidence in patients above the age of 70, the disease is more likely to affect female patients with a 5:1 ratio. Whereas in a pediatric minor group, male patients are more likely to be affected by the condition. This result is consistent with the low prevalence of congenital anatomical variants of the long gallbladder mesentery.

Traditionally, gallbladder volvulus has been diagnosed intraoperatively. As imaging technology improves and the number of preoperative imaging requests increases, the number of preoperative diagnosis reports rises, about 25% of cases in the last decade. Case reports and case series are the only sources of evidence for gallbladder volvulus. To minimize selection bias, further studies involving prospective cohorts or autopsy research might be beneficial. Nakao et al. reported a large series of 246 cases of gallbladder volvulus in 1999 [3], but only three of these were published in English literature; the remaining data were obtained from a Japanese national database. The only other available systematic review, published subsequently by Reilly et al., included 324 cases of gallbladder volvulus [5]. The lack of information and transparency about the included cases is a major limitation of their review, with a difference between the list of publications likely selected (n=521) and the total number of gallbladder volvulus retrieved cases (n=324), making the data difficult to interpret. Gallbladder volvulus is definitely an underdiagnosed condition that might incriminate clinicians. Doppler should be done if there is evidence of compromised blood flow from a cystic artery and gallbladder wall, which may help rule out gallbladder volvulus or gallbladder gangrene. Cholecystectomy should be performed as soon as gallbladder volvulus or gallbladder gangrene is suspected.

Tokyo Guidelines 2018 suggested that surgery be performed as soon as possible following admission and within 72 hours of onset [44]. Early cholecystectomy for cases within 72 hours of patient presentation or symptom onset was associated with lower mortality rates, complication rates, incidence of bile duct injury, and conversion to open surgery, according to a meta-analysis of case study reports [45]. We agree with this conclusion in order to avoid further consequences from both acute cholecystitis and gallbladder volvulus.

## Conclusions

Gallbladder volvulus is an uncommon but serious disease that needs immediate medical attention. Ultrasonography is the first imaging modality used to make a diagnosis, and Doppler should be used if there is a high suspicion of gallbladder volvulus. When a gallbladder volvulus is detected, prompt surgical treatment is very important to avoid the morbidity of gangrene, gallbladder rupture, and biliary peritonitis, all of which decrease survival.
